# Cholera: The Biography

**DOI:** 10.3201/eid1701.101580

**Published:** 2011-01

**Authors:** Kimberly Sessions Hagen

**Affiliations:** Author affiliation: Emory University, Atlanta, Georgia, USA

**Keywords:** Cholera, history, 19th century medicine, 20th century medicine, bacteria, book review

Cholera: The Biography, by Christopher Hamlin, joins Obesity, Asthma, Hysteria, Diabetes, Thalassaemia, and Down’s Syndrome as part of the Oxford University Press series Biographies of Diseases. As great a read as Tuberculosis (Biographies of Disease) (*1*), Hamlin’s book does an excellent job of treating a complex subject with scientific rigor while also being completely accessible to a lay audience. It includes a glossary of terms and suggestions for further reading. In short, I would highly recommend this book to anyone interested in learning more about the origins and history of a “disease” about which the more we know the less certain we become.

The word disease appears in quotation marks above because, as the book makes eminently clear, the concept and case definition of cholera are defined differently in different contexts. As is stated in Chapter 6, Cholera’s Last Laugh:

[Cholera] may not be (or may not always have been) a South Asian export; is caused by a group of genetically unstable organisms, whose main ecological niche is warm seas not the human intestine; is, as a clinical matter, indistinct from other severe diarrheas (including the old *cholera nostras*) and is often defined in clinical rather than bacteriological terms; is subject to seasonal and environmental factors, not merely to the presence of infected persons; is not exclusively, or often even predominantly, water-borne, and may not always be a fecal-oral disease; [and] is as much a political problem as ever.

Hamlin makes a clearly articulated argument that politics plays a large part in how the lay public and public health professionals perceive and respond to cholera. As this biography elucidates, cholera has historically not only driven individual destiny (Mary Lennox would never have left India for Mistlethwaite Manor had not her parents, her ayah, and most of the servants died of cholera in the first pages of Frances Hodgson Burnett’s book The Secret Garden), but it has also defined and reflected prevailing attitudes about the politics, economy, race relations, and social hierarchies of entire nations to an extent not matched until the advent of AIDS. In Chapter 3, Citizen Cholera, the reasons given for the Italian prime minister’s absolute denial of the 1911 cholera epidemic bear a chilling resemblance to the motivations behind South Africa’s President Mbeki’s 20th century denial of the AIDS epidemic.

The book ends on a cautionary note, stating that even in the face of modern public health interventions, climate change could trigger ever larger outbreaks as rising, warming seas expand the niche of *Vibrio cholera*. However, Hamlin’s final statement sums up both the central conundrum of cholera and the central satisfaction of cholera’s treatment in this book: “Cholera will not disappear nor cease to mean. A great challenge will be to respond to the meanings it is given.”
